# Effects of Metal Oxide Nanoparticles on Toll-Like Receptor mRNAs in Human Monocytes

**DOI:** 10.3390/nano10010127

**Published:** 2020-01-10

**Authors:** Vladislav A. Vasilichin, Sergey A. Tsymbal, Anna F. Fakhardo, Elizaveta I. Anastasova, Andrey S. Marchenko, Alexander A. Shtil, Vladimir V. Vinogradov, Elena I. Koshel

**Affiliations:** 1 International Institute Solution Chemistry of Advanced Materials and Technologies, ITMO University, 197101 St. Petersburg, Russia; vasilichin@scamt-itmo.ru (V.A.V.); zimbal@scamt-itmo.ru (S.A.T.); fakhardo@scamt-itmo.ru (A.F.F.); anastasova@scamt-itmo.ru (E.I.A.); marchenko@scamt-itmo.ru (A.S.M.); shtil@scamt-itmo.ru (A.A.S.); 2 Blokhin National Medical Research Center of Oncology, 115478 Moscow, Russia

**Keywords:** nanoparticles, toll-like receptors, cytotoxicity

## Abstract

For the widespread application of nanotechnology in biomedicine, it is necessary to obtain information about their safety. A critical problem is presented by the host immune responses to nanomaterials. It is assumed that the innate immune system plays a crucial role in the interaction of nanomaterials with the host organism. However, there are only fragmented data on the activation of innate immune system factors, such as toll-like receptors (TLRs), by some nanoparticles (NPs). In this study, we investigated TLRs’ activation by clinically relevant and promising NPs, such as Fe_3_O_4_, TiO_2_, ZnO, CuO, Ag_2_O, and AlOOH. Cytotoxicity and effects on innate immunity factors were studied in THP-1(Tohoku Hospital Pediatrics-1) cell culture. NPs caused an increase of TLR-4 and -6 expression, which was comparable with the LPS-induced level. This suggests that the studied NPs can stimulate the innate immune system response inside the host. The data obtained should be taken into account in future research and to create safe-by-design biomedical nanomaterials.

## 1. Introduction

Nanostructured materials are currently being exploited in a range of biomedical applications, including bioimaging, biosensing, diagnosis, and therapy [1,2,3,4,5,6,7,8,9]. Metal oxide nanoparticles (NPs) have gained considerable attention owing to their unique physical and chemical properties, and have broad applications in nanomedicine [10,11,12,13].

In particular, the iron oxide NPs, Fe_3_O_4_ (magnetite), are MRI contrast agents used for the imaging of cancer, and of cardiovascular and inflammatory diseases [14,15]. In addition, magnetite has been approved by the Food and Drug Administration (FDA) and the European Medicines Agency (EMA) for the treatment of iron deficiency anemia [16]. Titanium dioxide (TiO_2_) NPs are used for cleaning air and water, and are used in pigments, cosmetics, and skincare products [17]. Zinc oxide (ZnO) NPs are among the most commonly used nanomaterials, with a wide range of applications including biomedical imaging, drug and gene delivery, biosensing, and antibacterial and antifungal applications [18]. Copper oxide (CuO) has a high potential in the development of biosensors [19,20,21], antifouling coatings [22,23], and biocidal [24,25,26] and antitumor agents [27,28,29]. Boehmite (AlOOH) can be used as an adjuvant for vaccines [30,31]. Silver oxide (Ag_2_O) NPs have the potential for diagnostic biological probes [32], and antibacterial and anticancer agents [33,34,35].

After uptake into the body, NPs are recognized by the immune system, which can suppress or activate NPs [36,37,38]. It is supposed that even non-toxic NPs are able to alter a normal defense reaction toward toll-like receptor (TLR) ligands. TLRs are transmembrane proteins that recognize a wide range of “foreign” materials [39,40]. TLRs play a key role in the activation of innate immunity by recognizing specific molecular patterns. Compounds recognized by TLRs are primarily the components of microbial cells. Additionally, recent results provided evidence that TLRs can be activated by metal oxide NPs [41]. In particular, TiO_2_, ZnO, ZrO_2_, and silver NPs modulated the immune responses via TLRs [42,43]. NPs can trigger cytokine synthesis and pro-inflammatory responses [44]. TiO_2_ NPs, diamond NPs, and Pt NPs can activate pro-inflammatory cytokine production, dendritic cell maturation, and naive T cell activation and proliferation [45,46]. ZnO NPs increased the expression of IFN- ***γ ***(Interferon gamma), TNF- α (Tumor necrosis factor alpha), and IL-12 (Interleukin 12) in primary human immune cells [47]. TiO_2_ and SiO_2_ NPs can activate the NLRP3 inflammasome and IL-1 release in human macrophage cell line THP-1, and in lipopolysaccharide (LPS)-primed murine bone marrow-derived macrophages [48,49]. Thus, it becomes apparent that the interactions between NPs and immune cells emerge as an essential area of nanomedicine [50,51].

However, systematic research in this area is limited, leaving gaps in our understanding of the NPs’ effects on TLRs and the whole immune system. This study is aimed at the analysis of TLRs’ expression as induced by NPs. Cytotoxicity and the effects on the innate immunity factors were studied in THP-1 cell culture, expressing TLR-4 and -6. Most studies of the NPs’ ability to modulate inflammatory responses have focused on TLR-4-mediated inflammation [42,43,52]. In addition to TLR-4, many studies have revealed the effects of enhancing of TLR-6 expression. [53,54] Therefore, we focused on these two TLRs. We investigated NPs relevant to nanomedicine, that is, Fe_3_O_4_, TiO_2_, ZnO, CuO, Ag_2_O, and AlOOH. NPs increased the expression of TLR-4 and -6 to various extents. The most potent TLR inductors were AlOOH NPs. CuO and TiO_2_ NPs also stimulated the expression of both TLRs, but to lesser extents. The most unreactive were Fe_3_O_4_ NPs.

## 2. Materials and Methods

### 2.1. Chemicals

Titanium isopropoxide (≥99%), iron (II) chloride tetrahydrate (≥98.5%), iron (III) chloride hexahydrate (≥99%), copper sulfate pentahydrate, zinc nitrate hexahydrate, aluminum isopropoxide, and MTT powder were obtained from Sigma-Aldrich (St. Louis, MO, USA). Argentum nitrate and ammonia solution (25%) were purchased from LenReactiv (Saint-Petersburg, Russia). Deionized water was from Elix Essential 3UV, Millipore from Merck (Darmstadt, Hessen, Germany). Dimethyl sulfoxide (DMSO) was from VWR (VWR International, Radnor, PA, USA). The RNA extraction kit and the MMLV-RT kit were purchased from Evrogen (Moscow, Russia).

### 2.2. Synthesis of NP Sol

Fe_3_O_4_ NPs: 2.5 g FeCl_2_·4H_2_O and 5 g FeCl_3_·6H_2_O were dissolved in 100 mL of deionized water under constant stirring (500 rpm). Then, a 12 mL portion of aqueous ammonia solution was added dropwise to this solution under constant stirring (500 rpm) at room temperature for 3 min. Using a magnet, the formed magnetite precipitate was collected and washed with deionized water until neutral pH was attained. The washed black precipitate was mixed with 100 mL of deionized water and subjected to ultrasonic treatment (37 kHz, 110 W) under constant stirring (300 rpm). The duration of the ultrasonic treatment was 120 min.

TiO_2_ NPs: 3.9 mL titanium isopropoxide was injected into 50 mL deionized water, followed by heating to 85 °C and stirring at 500 rpm for 15 min. The obtained suspensions were treated with ultrasound (260 W, 60 Hz) and simultaneously stirred for 160 min (300 rpm). The prepared TiO_2_ sol was stored in plastic vials at room temperature.

ZnO NPs: Zinc oxide NPs were synthesized by the precipitation method. Zinc nitrate hexahydrate (3.0 g) was put into a small beaker and dissolved in 50 mL of distilled water to obtain 0.2 M concentration. The solution was heated to 91 °C under constant stirring (450 rpm), and 2 mL of 1 M NaOH were added, until a massive amount of white precipitate was obtained. It was centrifuged (8000 *g*, 10 min) and washed with water and ethanol. The final solution was prepared in water.

Ag_2_O NPs: The same approach was used for the synthesis of argentum oxide NPs. The solution of silver nitrate (0.1 M) was prepared in 50 mL of distilled H_2_O. It was heated to 98 °C under vigorous stirring, then 1 mL of 5 M NaOH was added until a large amount of black precipitate was obtained. After centrifugation and several washings in water and ethanol, Ag_2_O NPs were dissolved in water and dried in an oven at 60 °C for 24 h.

CuO NPs: Copper oxide NPs were synthesized as previously described by Phiwdang et al. [55]. Copper sulfate pentahydrate (1.25 g) was dissolved in 50 mL of distilled H_2_O, so the final concentration was 0.1 M. The solution was heated to 100 °C under constant stirring (450 rpm). Then, 2 mL of 5 M NaOH were added under vigorous stirring until the solution turned black. The obtained NPs were centrifuged and washed several times in water and ethanol. Dried copper oxide NPs were used for further characterization and concentration determination.

AlOOH NPs: Boehmite NPs were synthesized as previously described [56,57,58], where 2.2 g of Al(C_3_H_7_O)_3_ were added to 50 mL of deionized water at 90 °C, and a white precipitate was formed immediately. Before ultrasound treatment, the precipitate was kept at 90 °C under vigorous stirring for 15 min to complete the production of boehmite NPs and to complete the evaporation of the isopropanol formed during hydrolysis. The final suspension was ultrasonically treated for 2 h, after which a viscous sol was formed. The resulting sol was cooled to room temperature.

### 2.3. Characterization Techniques

The crystal phase and crystallinity of samples were studied by the X-ray diffraction (XRD) method (Rigaku SmartLab 3 diffractometer (Tokyo, Japan) of the engineering center of the Saint Petersburg State Technological Institute (Technical University)) using Cu-Kα irradiation (λ = 1.54 Å). Samples were scanned along 2θ in the range of 10–70° at 0.5 degrees/min. For XRD analysis, samples were dried at 120 °C for 4 h. For SEM analysis, the samples were dried in vacuo for 2 h and examined using a Tescan VEGA 3 scanning electron microscope (Brno, Czech Republic). The particle size and zeta potential in colloidal solutions were measured using a Photocor EPM/Photocor Compact Z (Moscow, Russia). The surface area, pore volume, and pore size distribution were investigated using Quantachrome Nova 1200e (Boynton Beach, FL, USA) by nitrogen adsorption at 77 K, and analyzed using the BET and BJH equations. Prior to analysis, all samples were degassed at 110 °C for 4 h.

### 2.4. MTT Assay

To evaluate the NPs’ cytotoxicity, the human monocytic THP-1 cells were maintained in RPMI 1640 (Biolot, Saint-Petersburg, Russia) supplemented with 10% fetal bovine serum (Gibco, Australia) and 50 μg/mL gentamycin (Biolot Saint-Petersburg, Russia) at 37 °C, 5% CO_2_. Cells at a logarithmic phase of growth were plated (5 × 10^3^/well) into 96-well plates and treated for 72 h with NPs resuspended directly in the culture medium to reach final concentrations of 0.23–30 µg/mL. The volume of added NPs from the stock in water was <5% of the total volume of the culture medium in the wells. After the completion of cell exposure, 20 µL MTT (3-(4,5-dimethylthiazol-2-yl)-2,5-diphenyltetrazolium bromide; 0.5 mg/mL) solution in saline buffer were added to each well for 1.5 h. Then the solution was aspirated, and formazan granules were dissolved in 200 μL of dimethyl sulfoxide. Optical density was measured at 570 nm on a Tecan Infinite 50 spectrophotometer (Mennedorf, Switzerland). Cell viability was calculated as the percentage of optical densities in wells, with each concentration of NPs normalized to the optical density of untreated cells (100%).

### 2.5. NPs’ Influence on TLR Gene Expression

Cells at the logarithmic phase of growth were plated (10^6^/well) into 6-well plates and treated for 24 h with NPs resuspended directly in the culture medium at final concentrations (according to the MTT tests) in which cell viability was >80%: for Fe_3_O_4_ and AlOOH, 30 μg/mL; for TiO_2_, 25 μg/mL; for Ag_2_O, 15 μg/mL; for CuO, 0.5 μg/mL; and for ZnO, 1 μg/mL. As a positive control for TLR signaling activation, we used lipopolysaccharide (LPS) (Sigma, St. Louis, Missouri, USA).

### 2.6. RNA Extraction, Reverse Transcription and Quantitative PCR

An RNA extraction kit ExtractRNA (Evrogen) was used to extract total RNA from THP-1 cells. Concentration and purity of total RNA obtained after the extraction were quantified based on the absorbance at 260 nm using a Nanophotometer (Implen, Munich, Germany). Reverse transcription was performed using an MMLV-RT kit containing reverse transcriptase (Evrogen, Moscow, Russia) with the addition of hexamer primers to obtain cDNA from the RNA template. Both total RNA extraction and reverse transcription were performed according to the manufacturer’s protocols. Quantitative real-time PCR with the fluorescent probe SYBR-Green was used to assess the TLRs’ mRNAs in response to NPs or LPS. To normalize the expression data, we used the housekeeping reference gene beta-2-microglobulin. PCR was performed using the qPCRmix-HS SYBR reagent kit (Evrogen Moscow, Russia). Primers were the following: TLR-4 Forward: GCTCTGCCTTCACTACAGGGACT, Reverse: CTGGGACACCACGACAATAACC; TLR-6 Forward: TGGGCTAACATTAGAGCCGC, Reverse: GGCATGAGGATAATGGAGGCA; beta-2-microglobulin Forward: GATGAGTATGCCTGCCGTGT, Reverse: TGCGGCATCTTCAAACCTCC. Primer sequences were selected using NCBI Primer-BLAST and synthesized in Evrogen. Real-time PCR was performed on a CFX Connect™ Real-Time PCR Detection System (BioRad Laboratories, Hercules, CA, USA). Relative quantification of gene expression was performed using the comparative Cq method of calculating threshold cycles of genes of interest. Relative gene expression data were normalized to an internal control [59]. The standard deviation was determined based on values from triplicate samples.

### 2.7. Statistics

Each experiment in mammalian cultures was carried out in triplicate. Data are presented as mean ± SD. Statistical analyses were performed using the Student’s *t*-test or Mann−Whitney test (Statistica 6, StatSoft Inc., Tulsa, OK, USA). Statistical significance was considered at *p* < 0.05.

## 3. Results and Discussion

### 3.1. Characterization of NPs

**Figure 1:**
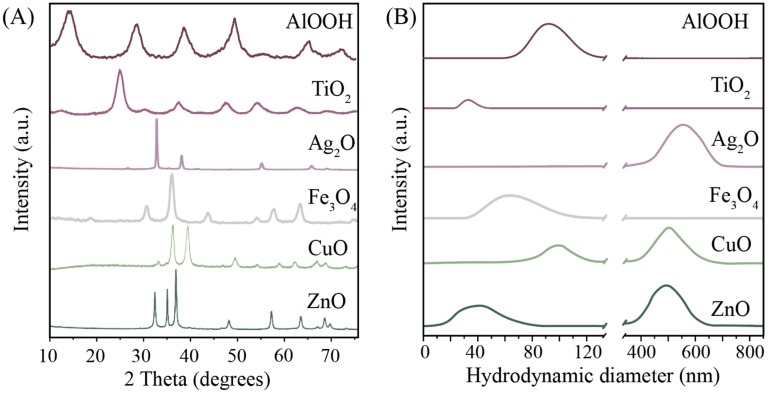
(**A**) X-ray diffraction (XRD) patterns of dried metal oxides; (**B**) DLS particle size distribution in the sols.

First, we obtained Fe_3_O_4_, TiO_2_, ZnO, Ag_2_O, CuO, and AlOOH NPs. The XRD analysis (Figure 1A) revealed that each material was highly crystalline. The XRD patterns were recorded within the fraction angle range of 10° to 70°. Thus, the XRD pattern of iron oxide showed that the main crystal phase is magnetite (JCPDS file No. 19-0629). Scherrer equation analysis indicated that the magnetite crystallites are of ~10 nm. Silver NPs revealed the diffraction peaks in the XRD pattern matched with Ag_2_O (JCPDS file No. 42-0874), with XRD peaks of 26° and 32° 2θ, which are the indicators of Ag_2_O NPs [60]. The characteristic peaks of copper oxide located at 2θ were equal to 32.58°, 35.47°, and 38.97° for CuO (JCPDS file No. 80-1268), while the main phase was tenorite. The XRD of zinc oxide showed broad peaks at values of 31.9°, 34.5°, 36.3°, and 62.0°, typical for the zinc oxide structure (JCPDS file No. 36-1451). In the case of titanium oxide, the main phase is anatase (JCPDS file No. 21-1272) with the crystallite size of 12 nm. All diffraction peaks in AlOOH samples were indicative of a boehmite (JCPDS file No. 21-1307) crystalline phase with a crystallite size of 6 nm.

The described procedures allowed us to obtain the hydrosols of metal NPs with various parameters. Determining the modality of hydrosols by dynamic light scattering (DLS), we found that the hydrosols of zinc and copper were characterized by a multimodal distribution (Figure 1B). This meant that these hydrosols were less stable compared to the sols of iron, titanium, and alumina oxides. In particular, the hydrodynamic diameter of the resulting particles was 40–600 nm. The reduced physical stability of the hydrosols of zinc, copper, and silver oxides can also be explained by the relatively low value of zeta potential (up to 15 mV modulo). Table 1 presents the values of zeta potential for the studied systems. One may see that the charge of the particles of iron, titanium, copper, zinc, and aluminum is positive, whereas silver NPs are negatively charged (ζ = −15 mV). It is worth noting that the charge does not affect the stability of aqueous solutions.

**Figure 2:**
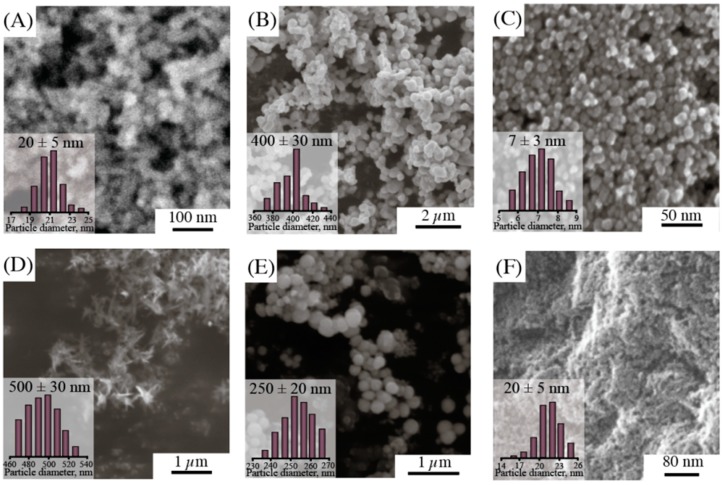
SEM images and particle size distribution by SEM of NPs: (**A**) TiO_2_, (**B**) Ag_2_O, (**C**) Fe_3_O_4_, (**D**) CuO, (**E**) ZnO, (**F**) AlOOH.

The structures of NPs were also analyzed using scanning electron microscopy (SEM; Figure 2). The particle size distribution was determined according to SEM. The samples of CuO were rods, which explained their large size and multimodal particle size distribution according to DLS. Other new NPs had a spherical shape, while their distribution was quite narrow. A comparison of size distributions by SEM for Ag_2_O and ZnO with DLS particle distribution demonstrated that the measured results are inconsistent. This fact can be related to particle agglomeration into large clusters.

**Figure 3:**
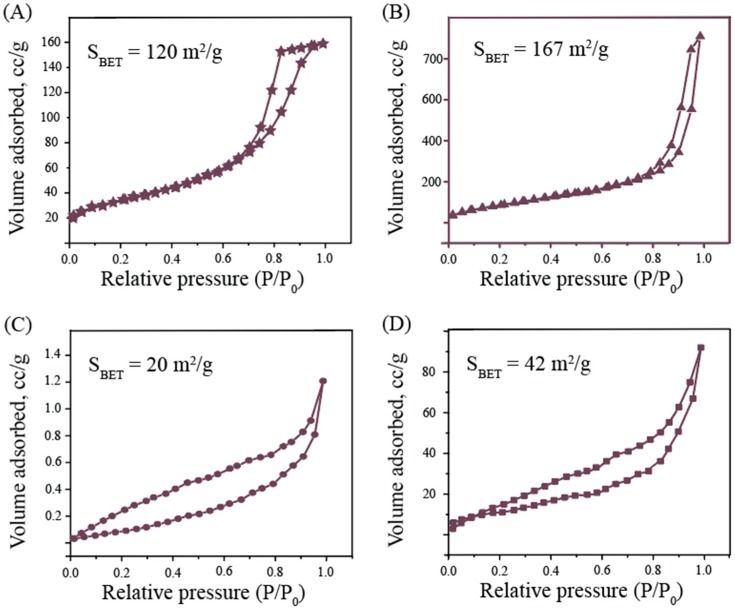

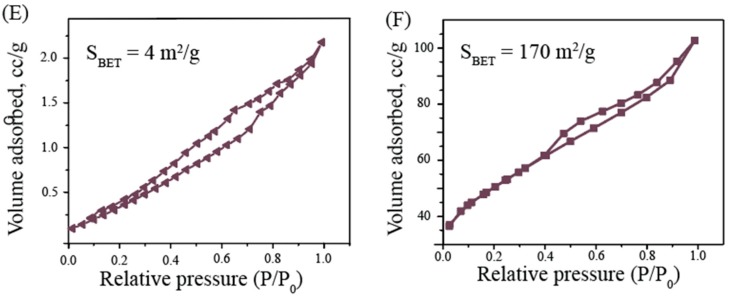
Characterization of samples by low-temperature physisorption. Shown are nitrogen adsorption–desorption isotherms of NPs: (**A**) Fe_3_O_4_, (**B**) TiO_2_, (**C**) ZnO, (**D**) CuO, (**E**) Ag_2_O, (**F**) AlOOH. The surface area for all samples was calculated by the BET method.

The surface of metal oxides by low-temperature nitrogen physisorption was studied (Table 1 and Figure 3). All newly synthesized materials were mesoporous with a narrow pore size distribution. The surface area calculated by BET was the largest for TiO_2_, Fe_3_O_4_, and AlOOH NPs (167, 120, and 170 m^2^/g, respectively), which is consistent with the initial sizes of the obtained particles.

### 3.2. Cytotoxicity of NPs

**Figure 4:**
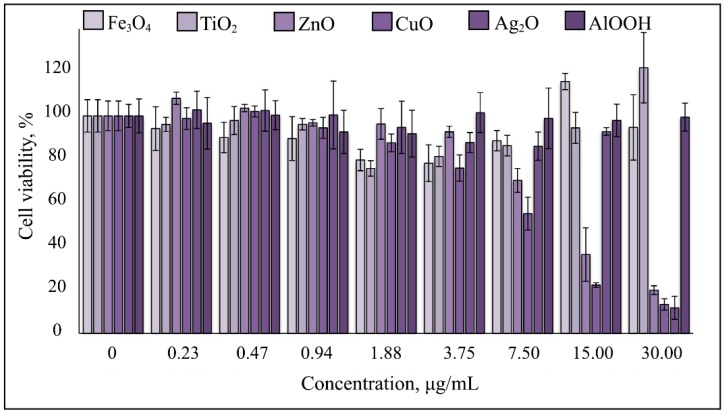
Cytotoxicity of Fe_3_O_4_, AlOOH, TiO_2_, Ag_2_O, CuO, and ZnO NPs after 72 h of exposure of the THP-1 monocyte cell line. Shown are the mean of three independent experiments (three replicates in each) ± standard deviation.

Fe_3_O_4_, TiO_2_, and AlOOH NPs evoked no cytotoxic effects on human monocytic cells (THP-1 line) at the range of concentrations of 0.234–30 μg/mL for at least 72 h of continuous exposure. The cell survival rate did not fall below 80%. No morphological signs of death were detectable, indicating that even the maximum concentrations used in this experiment were tolerable. Ag_2_O NPs were cytotoxic at 30 μg/mL, whereas at 0.234–15 μg/mL, cell viability was ≥80%. CuO and ZnO were toxic at ≥15 μg/mL (Figure 4). In general, the results of the cytotoxicity analysis coincide with the previously published results [61,62]. Using the results of this analysis, we have calculated the optimal NP concentrations to analyze their effects on TLR-4 and -6 expression.

### 3.3. NPs Enhance TLR-4 and TLR-6 mRNA Levels in THP-1 Cells

In this study, we aimed to demonstrate the immune system response caused by the exposure to NPs on the model of activation of TLRs in THP-1 monocytes. This study is relevant because the NPs used in the investigation have a high therapeutic potential and can be used to create new approaches to the treatment of diseases, such as bacterial and fungal infections, through the additional stimulation of the immune system [36,37,38]. TLRs are present in most cell types and are part of the signaling pathways that respond to various compounds; in particular, bacterial cell wall components such as lipopolysaccharide [39,40]. They cause the activation of cellular mechanisms that lead to increased activity of T and B cells, as well as macrophages. This provides an immune response [62].

To estimate the influence of NPs on TLR-4 and -6 mRNAs in THP-1 monocytes, we used the concentrations at which cell viability was at least 80% as determined in MTT tests. We accepted that the corresponding equipotent values for these viability levels were as follows: 30 μg/mL for Fe_3_O_4_ and AlOOH, 25 μg/mL for TiO_2_, 15 μg/mL for Ag_2_O, 1 μg/mL ZnO, and 0.5 μg/mL for CuO. Here, we incubated cells with these NPs or with 1μg/mL LPS as a positive inductor of TLR response for 24 h.

**Figure 5:**
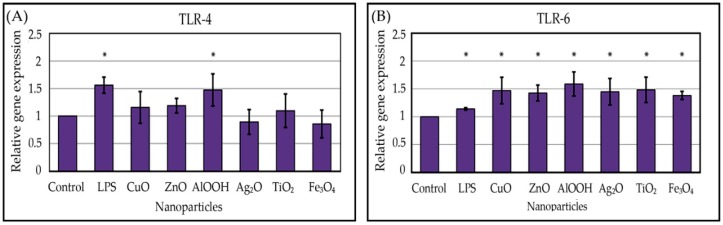
Relative expression of toll-like receptor 4 (TLR-4) (**A**) and TLR-6 (**B**) genes in THP-1 cells after 24 h exposure to NPs or lipopolysaccharide (LPS). Shown are the mean of three independent experiments (three replicates in each) ± standard deviation. See Materials and Methods for details. * *p* < 0.05.

All the studied NPs increased the TLRs’ expression to different degrees (Figure 5), however, the maximum induction was comparable with one after the LPS exposure. The maximum induction of TLR-4 was observed under the AlOOH NPs’ influence, which increased the expression 1.5-fold. Of note, the level of TLR-4 following AlOOH was comparable with that of LPS. CuO, ZnO, and TiO_2_ NPs increased the expression less significantly: 1.2, 1.2, and 1.1-fold, respectively. Ag_2_O and Fe_3_O_4_ did not affect the TLR-4 expression. TLR-6 was also the most induced by AlOOH NPs—1.6-fold. Among the other particles, Ag_2_O, TiO_2_, and CuO were more potent: fold induction values were 1.5, 1.5, and 1.5, respectively. The least comparative effects were produced by ZnO and Fe_3_O_4_ NPs, which increased the expression 1.4-fold. Thus, the most potent TLR inductor is AlOOH NPs. CuO and TiO_2_ NPs also stimulated the expression of both TLRs, but to a lesser extent. Silver did not affect the TLR-4 but instead strongly induced TLR-6. The most unreactive NPs were Fe_3_O_4_.

For some NPs we studied, immune response stimulation, including TLR-mediated stimulation, has been shown earlier [41,42,43,44,45,46,47,48,49]. In some cases, the TLR expression data we obtained were slightly lower than the previously published data. In particular, it has been shown that magnetite NPs specifically induced macrophage autophagy through activation of TLR-4 [16]. In our case, the Fe_3_O_4_ NPs did not affect the TLR-4. In addition, it has been shown that among the TLRs, TLR-6 was the most potent activator of inflammatory reactions induced by ZnO NPs [54]. In our study, they did activate the TLR-6 more strongly than the TLR-4, but in comparison with other NPs, their effect on the TLR-6 was one of the lowest. Such a discrepancy may be due to the peculiarities of NP synthesis, which causes their increased toxicity and immunogenicity, as well as the peculiarities of experiments on biological models. In this regard, it is especially important to conduct a systematic study of a set of NPs characterized and synthesized simultaneously in the same or similar conditions.

## 4. Conclusions

In this study, we analyzed the influence of metal oxide NPs on innate immunity by testing TLR-4 and -6 mRNAs in response to these nanomaterials in the human monocyte cell line. We detected no cytotoxicity for 72 h at 30 μg/mL for Fe_3_O_4_ and AlOOH, 25 μg/mL for TiO_2_, 15 μg/mL for Ag_2_O, 0.5 μg/mL for CuO, and 1 μg/mL for ZnO (the range of investigated concentrations was chosen according to the maximum NP concentration, forming stable sol in aqueous solution). All studied NPs activated TLR-6 expression, whereas AlOOH enhanced both TLR-4 and -6. Thus, the use of these NPs in vivo may have a dual effect, due to stimulation of the innate immune system. The effect may be beneficial due to the increased expression of anti-inflammatory cytokines. It is of particular importance for drug development against bacterial or fungal infections, where additional stimulation of the immune system can accelerate the antimicrobial response and tissue repair. However, these effects should be kept in mind when using these materials for anticancer drug development, since the attraction of immune cells to the tumor may be clinically unfavorable. At any rate, it should be noted that the maximum induction of expression was not very strong. The described results demonstrate only the potential effect of NPs on innate immunity, and emphasize the need for further research in this direction.

## Figures and Tables

**Table 1:** Hydrodynamic size, zeta potential, and surface parameters of nanoparticles (NPs).

Sample	Hydrosol Parameters		Surface Parameters	
	Hydrodynamic Diameter, nm	Zeta Potential, mV	S _BET_, m^2^/g	Pore Size, nm
TiO_2_	40 ± 7	+7.2 ± 0.3	167	5
Ag_2_O	510 ± 70	−16 ± 0.3	4	3.5
Fe_3_O_4_	60 ± 20	+30.0 ± 1.2	120	9
CuO	500 ± 50	+10.8 ± 0.4	42	3.3
ZnO	500 ± 70	+18.0 ± 0.3	20	3
AlOOH	90 ± 10	+42.0 ± 0.5	170	3.5
